# Integrated multi-omics analysis reveals distinct microbiota-metabolite signatures and a novel HCN2-2-hydroxybutyric acid interaction in inflammatory bowel disease

**DOI:** 10.3389/fnut.2026.1843166

**Published:** 2026-06-23

**Authors:** Menghui Zhang, Jingjing Jiang, Bo Yang, Wenzhuo Zhao, Jialing Zhang, Tianheng Ma, Honggang Wang

**Affiliations:** Department of Gastroenterology, The Affiliated Huaian No. 1 People's Hospital of Nanjing Medical University, Huai'an, Jiangsu Province, China

**Keywords:** 2-HB, gut microbiota, HCN2, inflammatory bowel disease, multi-omics, short-chain fatty acids

## Abstract

**Introduction:**

Gut microbiota-derived short-chain fatty acids (SCFAs) exert critical regulatory functions in inflammatory bowel disease (IBD). However, integrated profiling of fecal SCFA signatures alongside gut microbiota composition in ulcerative colitis (UC) and Crohn's disease (CD) remains insufficiently characterized. Furthermore, the molecular mechanisms through which microbiota metabolites engage host protein targets warrant systematic investigation.

**Methods:**

This study enrolled 30 patients with UC, 20 with CD, and 30 healthy controls, with paired fecal collection. Gut microbiota composition was analyzed by deep metagenomic sequencing, and SCFA concentrations were quantified by gas chromatography-mass spectrometry. Multi-omics integration, correlation network analysis, and Bayesian kernel machine regression were employed to resolve microbiota-metabolite associations. An integrated computational pipeline incorporating molecular dynamics simulations was constructed to evaluate the thermodynamic stability and binding modalities of metabolite-protein interactions.

**Results:**

Both UC and CD patients exhibited significantly reduced gut microbial α-diversity and characteristic community structure alterations. Fecal metabolomic profiling revealed synchronous elevation of 2-Hydroxybutyric acid (2-HB) and isocaproate in both patient groups, whereas butyrate reduction was restricted to UC. Multi-omics correlation analysis identified significant associations between 2-HB and unclassified Veillonella species as well as specific functional modules. Molecular dynamics simulations with an aggregate sampling time of 100 ns revealed a structural basis for the formation of a stable complex between 2-HB and the hyperpolarization-activated cyclic nucleotide-gated channel 2 (HCN2). This interaction was primarily mediated by electrostatic interactions involving Arg659, Arg618, and Arg617 residues alongside hydrophobic contacts, suggestive of potential allosteric modulation.

**Conclusions:**

This study identifies 2-HB and isocaproate as shared fecal metabolic markers across IBD and provides a structural rationale for the interaction between 2-HB and HCN2. The druggability profile of HCN2 supports its prioritization for mechanistic investigation, with the caveat that functional validation is prerequisite to any inference of therapeutic relevance.

## 1 Introduction

Inflammatory bowel disease (IBD), encompassing ulcerative colitis (UC) and Crohn's disease (CD), represents a chronic relapsing disorder of the gastrointestinal tract with increasing global incidence (1, 2). Although advances in clinical therapies continue, a substantial proportion of patients exhibit inadequate or non-response. There is an urgent need for non-invasive biomarkers to guide diagnosis and monitor treatment response. Current clinical research relies heavily on serological markers and endoscopic assessment, which are either invasive or lack specificity for distinguishing UC from CD (3, 4). Consequently, growing interest has emerged in exploring associations between gut microbiota and metabolism, which may serve as novel diagnostic and prognostic indicators.

Increasing evidence demonstrates that gut microbiota dysbiosis constitutes a hallmark of IBD pathogenesis (5–7). Compared with healthy individuals, both UC and CD patients exhibit reduced microbial diversity (8–10). Nevertheless, UC and CD exhibit distinct microbial signatures, potentially reflecting differences in disease location, lifestyle patterns, and intestinal barrier function (11, 12). However, numerous studies have focused on single-disease comparisons with controls; comprehensive analyses directly contrasting microbiome profiles of UC and CD within a unified study framework remain limited.

Short-chain fatty acids (SCFAs) are defined as fatty acids with fewer than six carbon atoms, primarily including acetic acid, propionic acid, isobutyric acid, butyric acid, isovaleric acid, valeric acid, and caproic acid. Short-chain hydroxy acids are also broadly considered a subgroup of SCFAs (13). These compounds have recently been intensively studied and have shown beneficial effects in various diseases. SCFAs are the principal metabolic products of bacterial fermentation of dietary fiber (14). SCFAs serve not only as energy substrates for colonocytes but also exert potent anti-inflammatory effects through specific signaling pathways, thereby maintaining intestinal homeostasis (14–16). Recent studies have documented reduced fecal SCFA concentrations in IBD patients, correlating with disease activity and microbial dysbiosis (17, 18). SCFAs are absorbed across the colonic epithelium into systemic circulation, suggesting that the concentrations of SCFA in feces may serve as indicators of both microbial metabolic function and intestinal barrier integrity (19).

Extensive research has investigated individual assessments of gut microbiota or SCFA metabolism. Nevertheless, such approaches capture only partial aspects of the host-microbe metabolic axis. Few studies have simultaneously characterized the gut microbiota, fecal SCFA profiles and the network pharmacology in IBD. Integrative multi-omics strategies, combining gut microbiota composition with functional metabolic readouts, may offer a more holistic understanding of IBD pathophysiology. Such approaches may identify microbial taxa functionally linked to SCFA perturbations, discriminate UC from CD with enhanced accuracy, and reveal novel therapeutic targets. However, integrated multi-omic analyses of gut microbiome and fecal SCFAs in well-characterized cohorts comprising healthy controls, UC, and CD patients are currently lacking. The present study performs a comprehensive multi-omic analysis of gut microbiota and SCFA profiles across these three groups, followed by network pharmacology and molecular docking. These findings may provide novel insights into the metabolic consequences of gut dysbiosis in IBD and identify potential biomarkers for clinical application.

## 2 Materials and methods

### 2.1 Study cohort and sample collection

This case-control study enrolled 30 patients with active ulcerative colitis (UC), 20 patients with active Crohn's disease (CD), and 30 healthy controls (Con) at the Affiliated Huaian No. 1 People's Hospital of Nanjing Medical University. Healthy controls were recruited from individuals undergoing routine health screening with no history of gastrointestinal disease, autoimmune disorders, or chronic inflammatory conditions. Exclusion criteria for all participants included: (i) antibiotic or probiotic use within 4 weeks prior to sampling; (ii) infectious enterocolitis within 3 months; (iii) prior intestinal resection or ostomy; (iv) diagnosis of indeterminate colitis; and (v) pregnancy or lactation. In the present cohort, 90% (45/50) of the patients have used mesalamines, glucocorticoids, biological agents or small molecule drugs in the past, but they were currently in the active stage of the disease after specialist evaluation. Written informed consent was obtained from all participants. The study protocol was approved by the Ethics Committee of the Affiliated Huaian No. 1 People's Hospital of Nanjing Medical University (Approval No: KY-2026-029-01), and conducted in accordance with the Declaration of Helsinki.

### 2.2 Metagenomic sequencing and microbiota analysis

Fresh fecal samples were collected in sterile containers, immediately aliquoted into cryovials, and flash-frozen in liquid nitrogen within 30 min of collection. All samples were maintained at −80 °C until analysis. Fecal DNA was extracted using the DNeasy PowerSoil Pro Kit (Qiagen), quantified by Qubit 4.0, and sequenced on the Illumina NovaSeq 6000 platform (150 bp paired-end reads, 8 Gb per sample). Raw reads were quality-filtered (fastp v0.23.2), depleted of host sequences (Bowtie2 v2.4.5), and taxonomically classified using Kraken2 (v2.1.2) with the PlusPF database. Relative abundances were computed by Bracken (v2.7). Alpha diversity (Shannon, Simpson indices) and beta diversity (Bray-Curtis dissimilarity, principal coordinate analysis) were analyzed at the species level using R packages vegan and phyloseq. Differential abundance was assessed by DESeq2 (v1.38.0) with Benjamini-Hochberg FDR correction.

### 2.3 UPLC-MS/MS

Fecal metabolites were extracted by homogenizing ~5 mg material with zirconia beads in 25 μL water and 120 μL methanol (internal standards), centrifuged at 18,000 × g (20 min, 4 °C), derivatized (20 μL supernatant, 30 °C, 60 min), diluted with 330 μL 50% methanol, and recentrifuged at 4,000 × g (30 min, 4 °C). Separation was performed on an ACQUITY UPLC BEH C18 column (2.1 × 100 mm, 1.7 μm) at 40 °C, 0.4 mL/min, with mobile phases A (0.1% formic acid in water) and B (acetonitrile-isopropanol, 70:30). Gradient: 0–1 min, 5% B; 1–11 min, 5%−78% B; 11–13.5 min, 78%−95% B; 13.5–14 min, 95%-100% B; 14–16 min, 100% B; 16–16.1 min, 100%−5% B; 16.1–18 min, 5% B. Analysis was performed on an ACQUITY UPLC-Xevo TQ-S system (5 μL injection). Data were processed by automated peak integration and isotope-dilution quantification. Metabolites with coefficient of variation > 15% across quality controls were excluded. Concentrations are reported as μg/g feces.

### 2.4 Multi-omics integration and statistical analysis

Statistical analyses were performed using SPSS 26.0 and R 4.2.1. Continuous variables are presented as mean ± SD or median (IQR); categorical variables as frequency (%). Group comparisons used ANOVA, Kruskal-Wallis, or chi-square tests as appropriate. Linear discriminant analysis Effect Size (LEfSe) analysis was performed to identify species with significantly different abundances among groups. First, the Kruskal-Wallis rank was used to assess differences in species abundance across groups. Features that passed the significance threshold were then subjected to linear discriminant analysis (LDA) to estimate the effect size. Only taxa with an absolute LDA score greater than 2.0 were retained. Spearman correlation assessed associations between microbiota, SCFAs, and clinical parameters (|rho| > 0.3, FDR < 0.05). Multi-omics integration employed sparse partial least squares discriminant analysis (sPLS-DA; mixOmics). Bayesian kernel machine regression (BKMR) estimated joint SCFA effects on IBD, with 50,000 MCMC iterations and covariate adjustment.

### 2.5 Compound target prediction and molecular dynamics simulation

An integrated five-module pipeline was developed: (i) human protein database construction from UniProtKB (UP000005640); (ii) druggability scoring (Drugged Score and Fpocket v4.2.2 structural analysis); (iii) multimodal feature extraction (TAPE tokenizer for proteins, RDKit and graph neural networks for small molecules); (iv) compound-protein interaction prediction using pre-trained TransformerCPI2.0; and (v) weighted ensemble ranking (0.3 × Drugged + 0.2 × Druggability + 0.5 × CPI). Top-ranked targets were selected for validation. The HCN2-2-HB complex was simulated using GROMACS 2020.6 (AMBER99SB-ILDN force field, TIP3P water, 150 mM NaCl). Following energy minimization and equilibration (NVT 100 ps, NPT 1 ns), production runs of 100 ns were performed at 310 K, 1 bar. Trajectory analysis included RMSD, RMSF, radius of gyration, hydrogen bond occupancy, and binding free energy estimation (MM/PBSA). Visualizations used PyMOL 2.5.

## 3 Results

### 3.1 Gut microbial alterations in IBD

**Figure 1:**
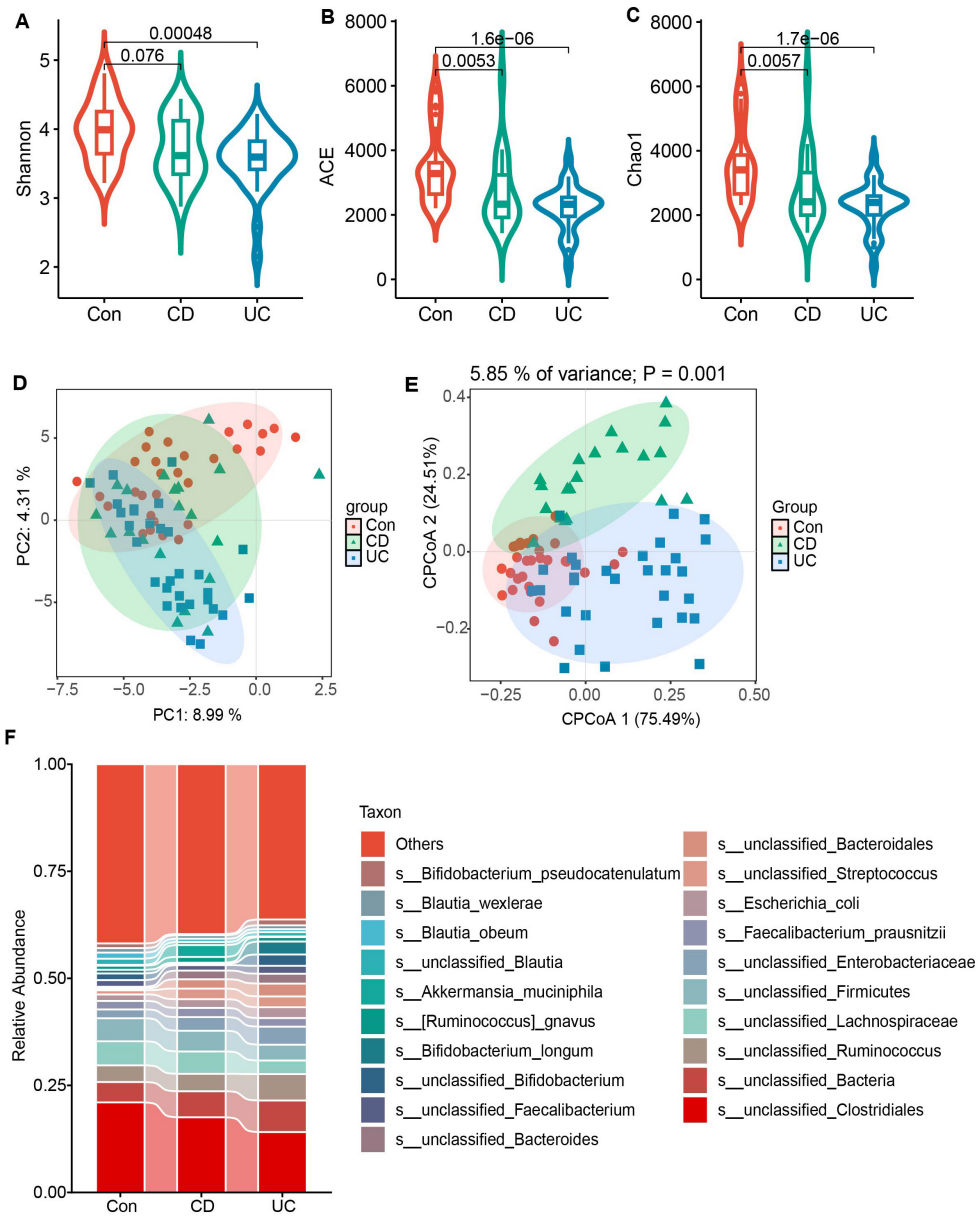
Alterations of gut microbial constructions in IBD. Comparison of α-diversity by **C** Chao1 and **(A, B)** Shannon indices using ANOVA in patients with ulcerative colitis (UC), Crohn's disease CD and healthy controls (Con). **(D)** Principal Component Analysis (PCA) and **(E)** Canonical Principal Coordinate Analysis (CPCoA) showed a significant difference in the gut microbial composition among UC, CD groups and Con. **(F)** The distribution of top 20 species detected in different phenotypic subgroups.

**Figure 2:**
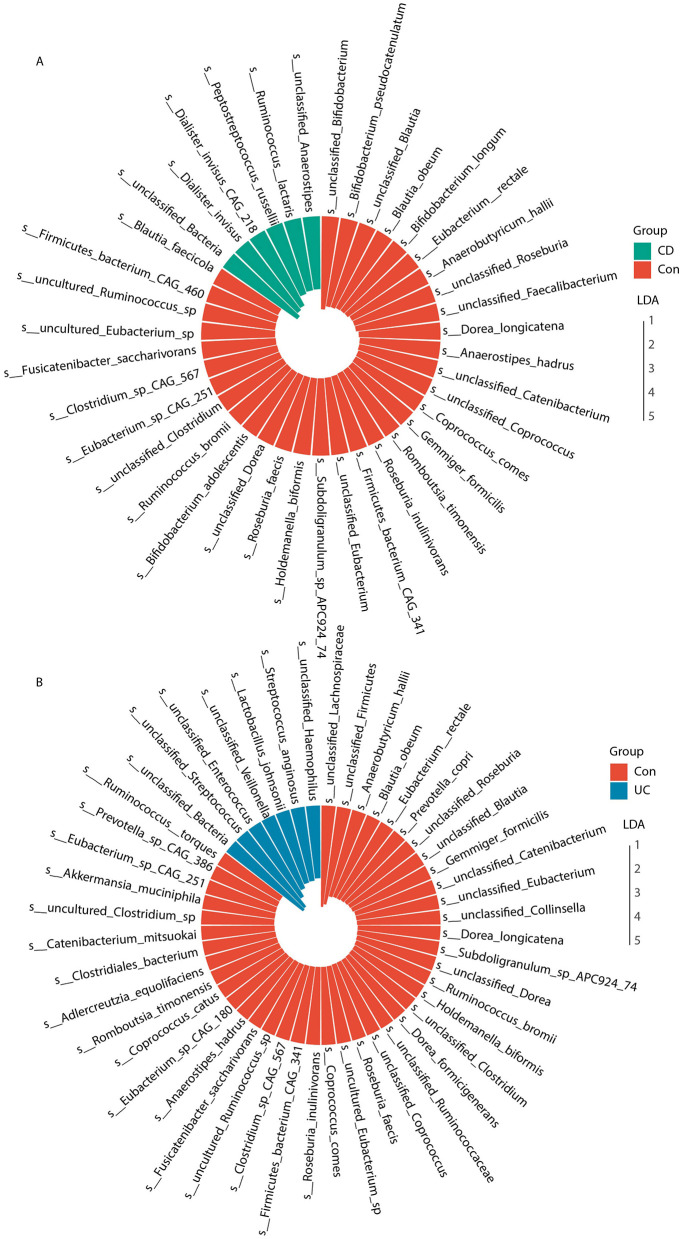
Alteration of gut microbiota by UC, CD and Con. **(A)** Linear discriminant analysis (LDA) scores of the gut microbiota in CD and Con. **(B)** LDA scores of the gut microbiota in UC and Con.

Alpha diversity was reduced in both UC and CD compared with Con, as measured by Chao1, ACE, and Shannon indices (Figures 1A–C). Principal component analysis (PCA) and constrained principal coordinate analysis (CPCoA) based on Bray-Curtis distances revealed distinct microbial community structures among the three groups (*P* = 0.001), indicating disease-specific alterations in gut microbiota composition (Figures 1D, E). The top 20 species showed differential distribution across phenotypic subgroups (Figure 1F). At the species level, *unclassified_Clostridiales* and *unclassified_Lachnospiraceae* exhibited progressive depletion from Con to CD to UC, whereas *unclassified_Ruminococcus* abundance increased in UC (Figure 1F). LEfSe identified disease-specific microbial signatures. CD patients showed enrichment of *Dialister_invisus, Dialister_invisus_CAG_218, Peptostreptococcus_russellii, Ruminococcus_lactaris, unclassied_ Bacteria*, and *unclassied_Anaerostipes* (Figure 2A). UC patients were characterized by enrichment of *unclassied_Bacteria, Streptococcus_anginosus, unclassied_Enterococcus, unclassied_ Veillonella, Lactobacillus_johnsonii, Streptococcus_anginosus*, and *unclassied_Haemophilus* (Figure 2B).

### 3.2 Fecal SCFA alterations in IBD

**Figure 3:**
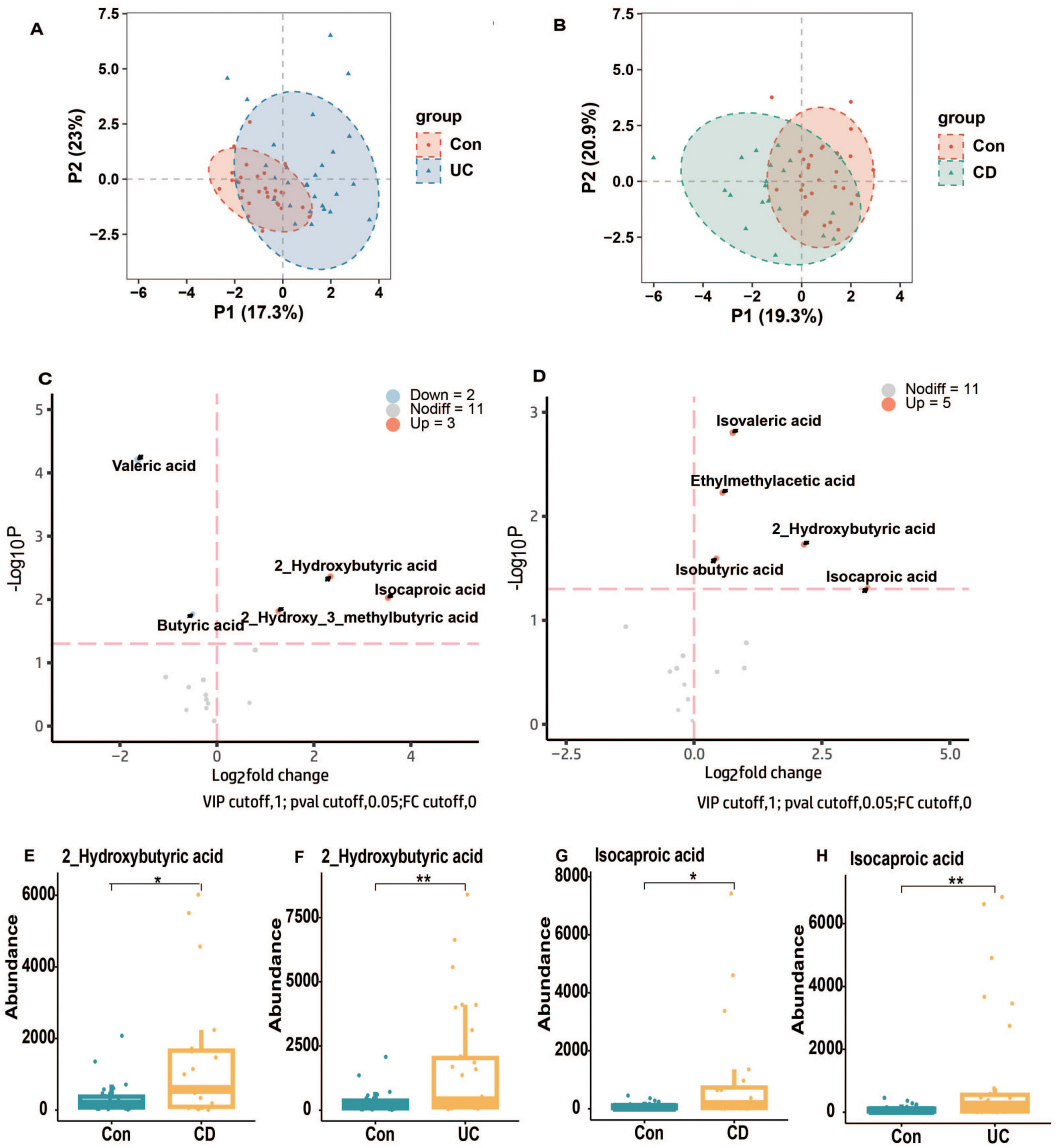
Changes in short-chain fatty acids (SCFAs) of the intestinal microbiota. Principal component analysis (PCA) showing the differences in intestinal metabolites between the control group and the UC **(A)** and CD **(B)** groups. The Volcano Plot illustrates the changes in SCFAs in the intestines of the UC group **(C)** and the CD group **(D)** compared to the control group. The differences of 2-HB between the control group and the CD and UC groups respectively **(E, F)**. The differences of isocaproic acid between the control group and the CD and UC groups respectively **(G, H)**.

PCA of fecal SCFA profiles demonstrated clear separation between CD and Con groups, with partial overlap between UC and Con, indicating more pronounced metabolic deviation in CD (Figures 3A, B). Compared with Con, UC patients showed significant alterations in four SCFAs: butyric acid and valeric acid were decreased, whereas isocaproic acid and 2-HB were increased (Figure 3C). CD patients exhibited upregulation of five SCFAs: isovaleric acid, ethylmethylacetic acid, 2-HB, isobutyric acid, and isocaproic acid (Figure 3D). Notably, 2-HB and isocaproic acid were concordantly elevated in both UC and CD, representing IBD-associated metabolic markers (Figures 3E–H).

### 3.3 Microbiota-SCFA correlations in IBD

**Figure 4:**
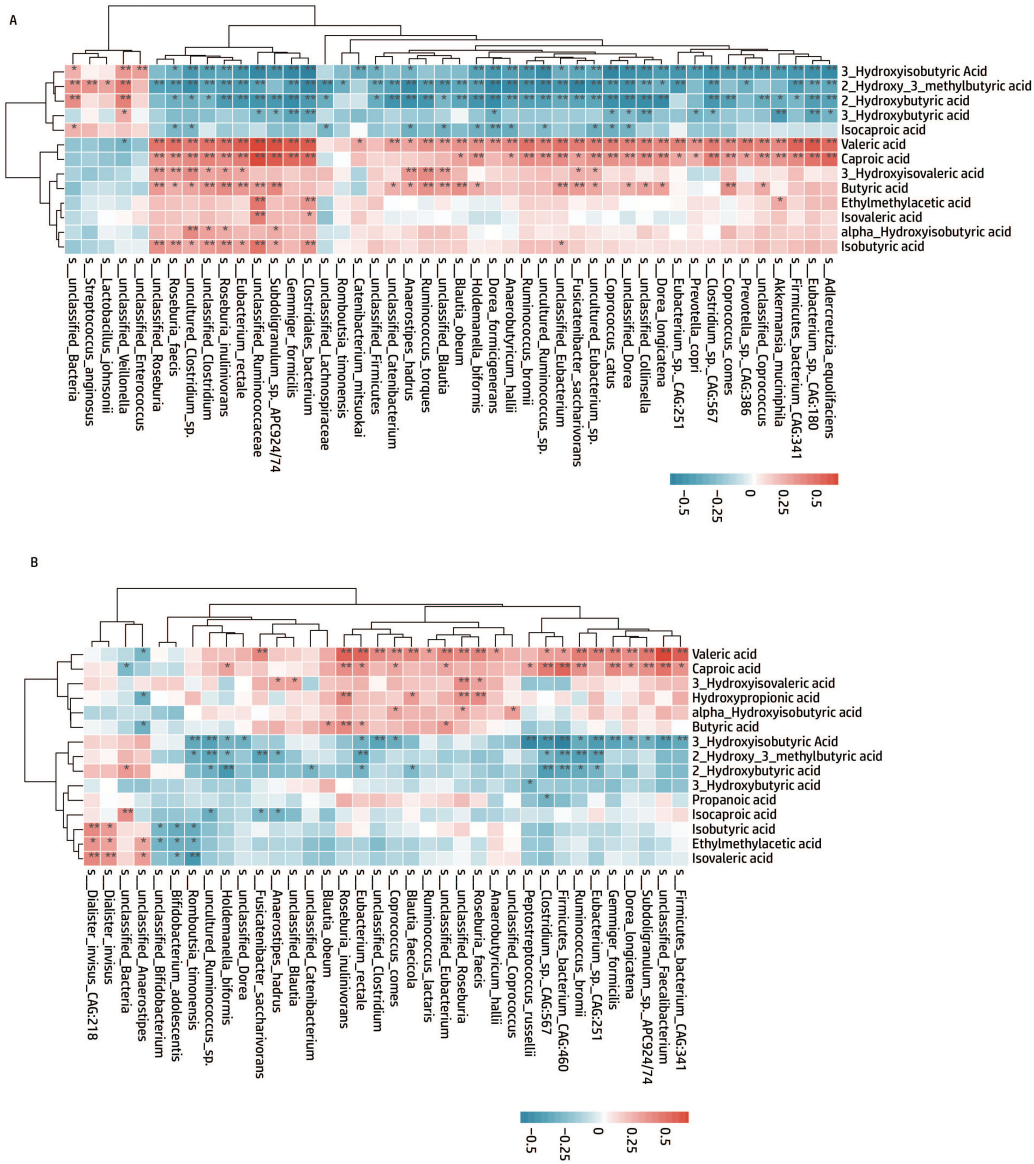
Correlations between the microbial species and SCFAs. A correlation analysis was conducted between the differential bacterial communities and SCFAs in UC **(A)** and CD **(B)** groups. The heat map shows Spearman correlation coefficients with significance indicated by ^*^P < 0.05, ^**^P < 0.01.

Correlation analysis between differential bacterial taxa and SCFAs revealed disease-specific associations. In UC, 2-HB positively correlated with *unclassied_Veillonella* and negatively correlated with multiple taxa (Figure 4A). Isocaproic acid negatively correlated with *Dorea_formicigenerans*. In CD, 2-HB negatively correlated with *Clostridium_sp._CAG:567, Firmicutes_bacterium_CAG:460*, and *Holdemanella_biformis* (Figure 4B). Valeric acid and caproic acid showed consistent positive associations with multiple bacterial taxa in both diseases.

### 3.4 BKMR analysis of SCFA mixture effects

**Figure 5:**
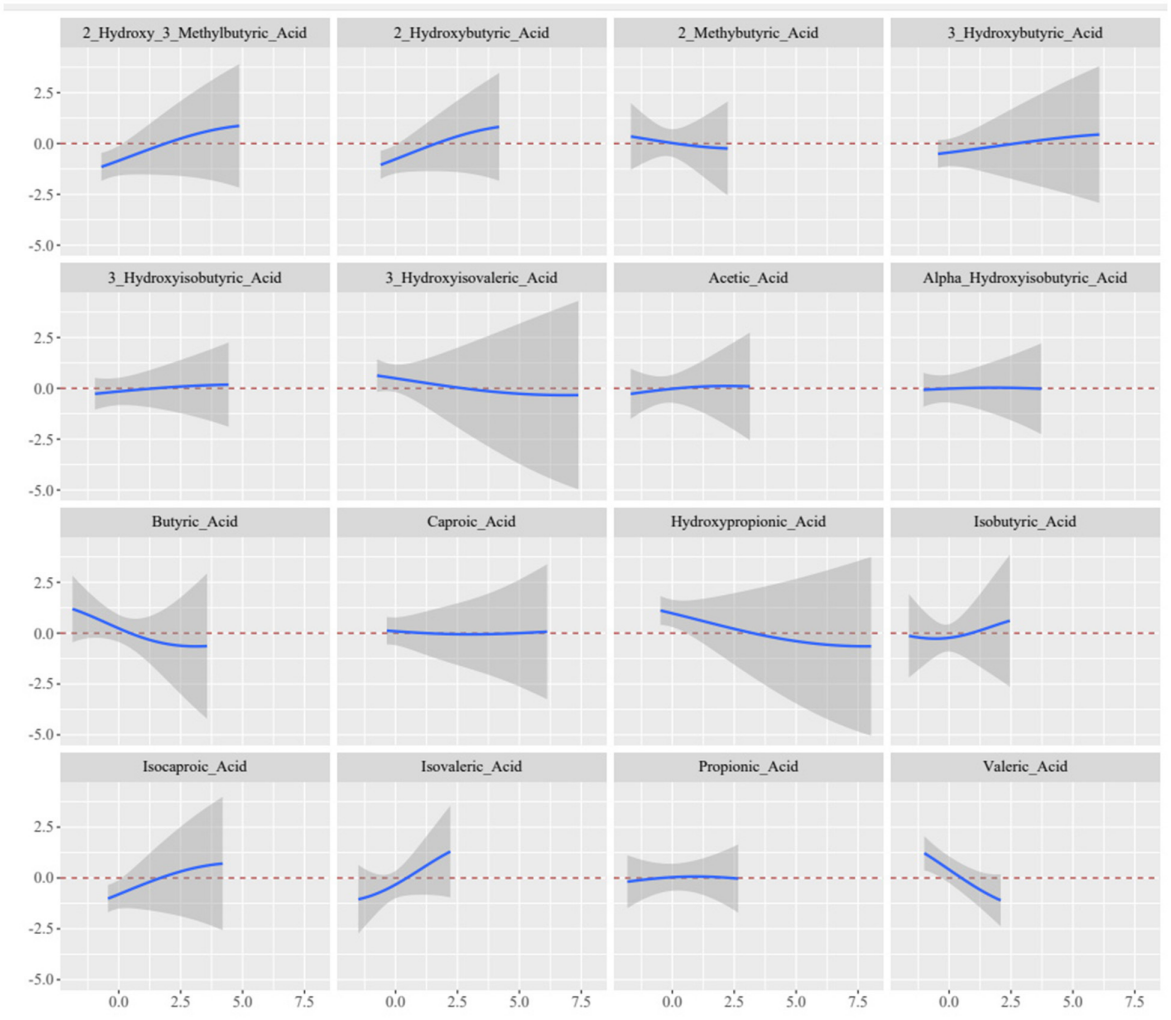
The BKMR model predicts the comprehensive evaluation effect of SCFAs on inflammatory bowel disease.

Bayesian kernel machine regression (BKMR) identified 2-HB and isocaproic acid as primary drivers of IBD risk, with elevated exposure levels associated with increased disease probability. Straight-chain SCFAs (acetic acid, butyric acid, propionic acid) showed no independent effects in the mixture model (Figure 5).

### 3.5 Ion channels dominate the top-tier predicted therapeutic targets

Based on the molecular target prediction analysis for 2-HB, this study systematically screened potential human protein targets by integrating Druggability Score and Compound-Protein Interaction (CPI) Score through an ensemble scoring strategy. The top ten candidate targets with the highest ensemble scores are presented in Table 1, with HCN2 (Ensemble Score: 0.941), ESR2 (0.928), PPARA (0.924), RXRA (0.914), and PARP1 (0.900) ranking as the top five, demonstrating exceptional comprehensive target potential. Notably, HCN2, a hyperpolarization-activated cyclic nucleotide-gated potassium channel subunit, exhibited balanced performance across both druggability (0.918) and CPI (0.915) dimensions. PPARA achieved the highest druggability score (0.961) among all candidates, indicating optimal drug-like properties, whereas ESR2 displayed the strongest ligand-binding potential with the highest CPI score (0.929). The candidate list was notably enriched with nuclear receptor family members (PPARA, RXRA, RXRG) and ion channel proteins (HCN2, HCN1, KCNMA1, KCNH8), suggesting that metabolic regulation and electrophysiological function-related pathways may play significant roles in the underlying disease mechanisms. These target prediction results provide data-driven support and directional guidance for subsequent drug design and functional validation studies.

**Table 1:** Top 10 candidate therapeutic targets identified by ensemble scoring strategy.

Protein	Symbol	Druggability score	CPI score	Ensemble score
Q9UL51	HCN2	0.918	0.915	0.941
Q92731	ESR2	0.820	0.929	0.928
Q07869	PPARA	0.961	0.865	0.924
P19793	RXRA	0.956	0.846	0.914
P09874	PARP1	0.732	0.907	0.900
O60741	HCN1	0.873	0.832	0.890
Q12791	KCNMA1	0.953	0.800	0.890
Q96L42	KCNH8	0.848	0.831	0.885
P08172	CHRM2	0.887	0.807	0.881
P48443	RXRG	0.953	0.773	0.877

Protein targets are ranked in descending order based on the Ensemble Score, which integrates Druggability Score (predicted likelihood of the protein being modulated by drug-like small molecules) and CPI Score (predicted compound-protein interaction potential). Protein IDs correspond to UniProt accession numbers. HCN2, hyperpolarization-activated cyclic nucleotide-gated potassium channel 2; ESR2, estrogen receptor 2; PPARA, peroxisome proliferator-activated receptor alpha; RXRA, retinoid X receptor alpha; PARP1, poly(ADP-ribose) polymerase 1; HCN1, hyperpolarization-activated cyclic nucleotide-gated potassium channel 1; KCNMA1, potassium calcium-activated channel subfamily M alpha 1; KCNH8, potassium voltage-gated channel subfamily H member 8; CHRM2, cholinergic receptor muscarinic 2; RXRG, retinoid X receptor gamma.

### 3.6 Dynamic and structural stability analysis of the HCN2-2-HB complex

**Figure 6:**
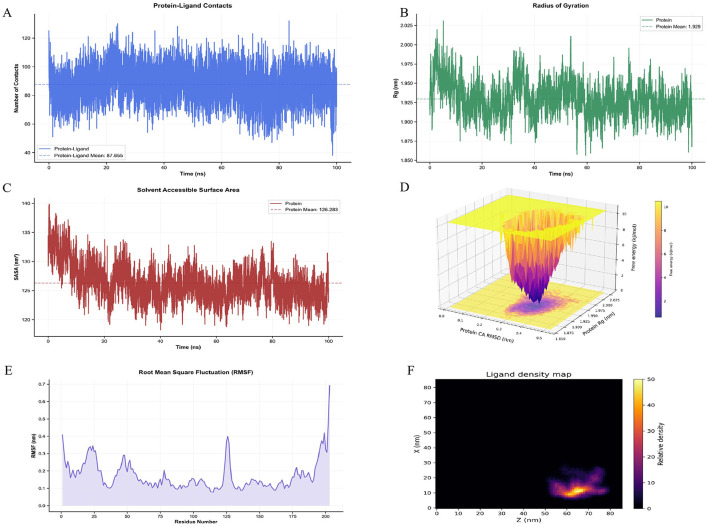
Dynamic and structural stability analysis of the HCN2-2-HB complex. **(A)** Protein-ligand contact number over 100ns simulation (mean = 87.7). **(B)** Radius of gyration of HCN2 (mean = 1.929 nm). **(C)** Solvent-accessible surface area (mean = 126.3 nm^2^). **(D)** Free energy landscape as a function of Cα RMSD and radius of gyration. **(E)** Root-mean-square fluctuation of Cα atoms. **(F)** Spatial density distribution of 2-HB in the binding pocket. Dashed lines in **(A–C)** indicate average values.

The 100 ns MD simulation revealed stable binding of 2-HB to HCN2 with minimal structural perturbation to the protein. The protein-ligand contact number remained consistently high throughout the simulation, averaging 87.7 ± 15.2 contacts, indicating persistent intermolecular interactions (Figure 6A). The radius of gyration of HCN2 stabilized at 1.929 ± 0.025 nm after initial equilibration, with no significant drift or expansion observed (Figure 6B). The solvent-accessible surface area fluctuated around 126.3 ± 3.5 nm^2^, further confirming the structural integrity of the protein-ligand complex (Figure 6C). The free energy landscape analysis identified a single dominant low-energy minimum at Cα RMSD ≈ 0.25 nm and Rg ≈ 1.93 nm, suggesting that the system predominantly samples a well-defined bound conformation without substantial conformational transitions (Figure 6D). RMSF analysis demonstrated that the N-terminal (residues 1–25) and C-terminal (residues 175–200) regions exhibit higher flexibility, with peak RMSF values reaching 0.41 nm and 0.69 nm, respectively, whereas the central domain (residues 75–125) remains relatively rigid with RMSF values below 0.15 nm (Figure 6E). Notably, residue 125 displays a pronounced flexibility peak, which may be functionally relevant for channel gating. The ligand density map illustrates that 2-HB is tightly localized within a specific binding pocket, with the highest density concentrated at X ≈ 10–25 nm and Z ≈ 55–75 nm coordinates, indicating stable and specific binding without significant ligand migration or dissociation events (Figure 6F).

### 3.7 Interaction analysis and binding mode of 2-HB with HCN2

**Figure 7:**
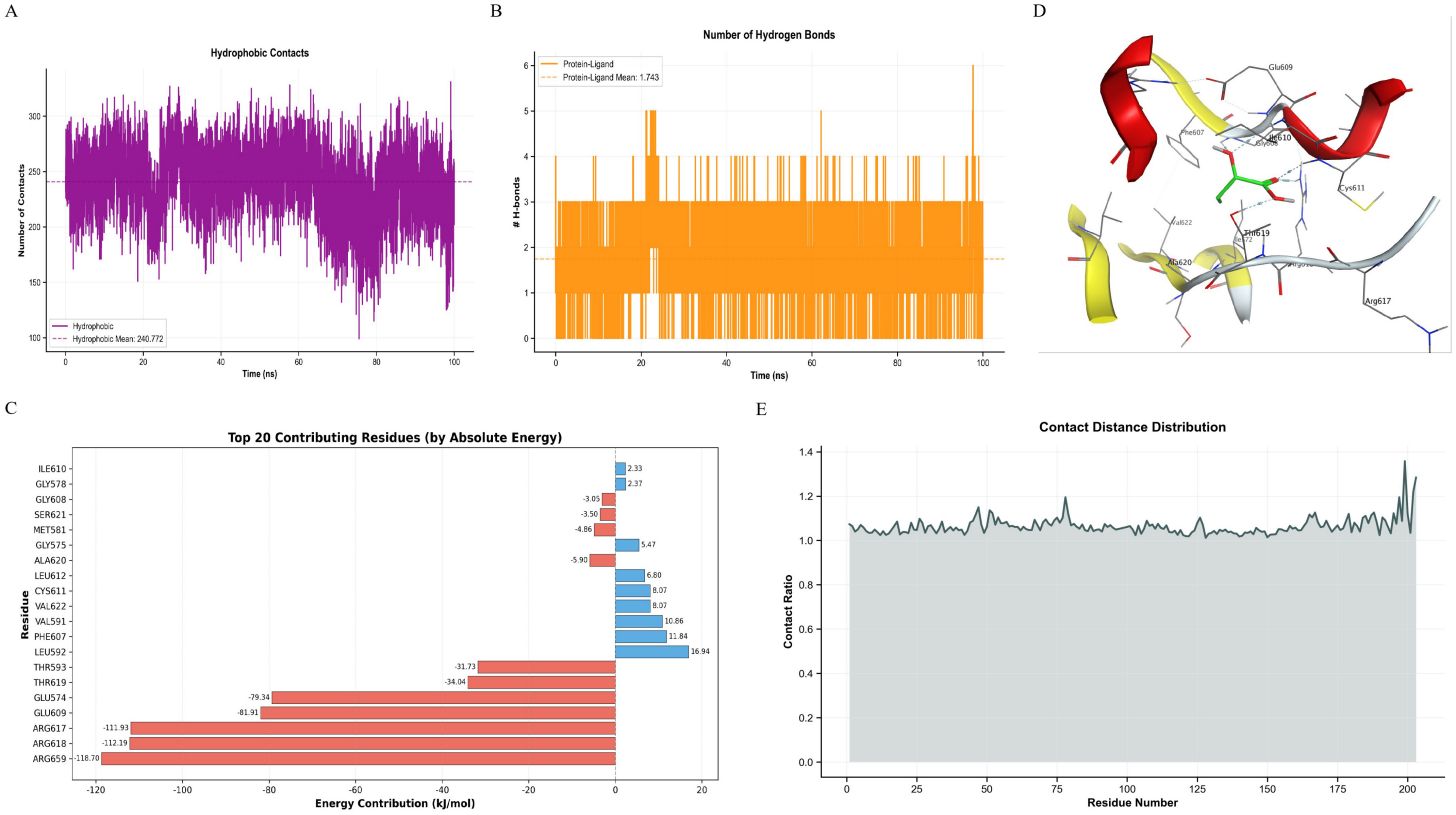
Interaction analysis and binding mode of 2-HB with HCN2. **(A)** Hydrophobic contact number over time (mean = 240.8). **(B)** Number of hydrogen bonds between protein and ligand (mean = 1.74). **(C)** Three-dimensional binding mode of 2-HB (green sticks) with key interacting residues labeled. **(D)** Top 20 residues ranked by absolute binding energy contribution (red: favorable; blue: unfavorable). **(E)** Contact distance distribution across protein residues. Dashed lines in **(A, B)** indicate average values.

Interaction analysis revealed that hydrophobic contacts constitute the predominant binding force, with an average of 240.8 ± 35.4 contacts maintained throughout the simulation (Figure 7A). Hydrogen bond analysis showed an average of 1.7 ± 0.9 hydrogen bonds between 2-HB and HCN2, with occasional formation of up to 5–6 hydrogen bonds during specific simulation intervals (Figure 7B). The three-dimensional binding mode visualization confirms that 2-HB is surrounded by Phe607, Gly608, Ile610, Cys611, Glu609, Thr619, Arg617, and Arg618, forming an extensive interaction network that stabilizes the ligand in the binding pocket (Figure 7C). Energy decomposition analysis identified three arginine residues-Arg659, Arg618, and Arg617-as the primary contributors to binding affinity, with energy contributions of −118.70, −112.19, and −111.93 kJ/mol, respectively (Figure 7D). These residues, together with Glu609 (−81.91 kJ/mol) and Glu574 (−79.34 kJ/mol), form a positively charged recognition pocket that anchors the carboxyl group of 2-HB through strong electrostatic interactions and hydrogen bonding. The contact distance distribution across protein residues showed elevated contact ratios at the C-terminal region (residues 190–200), consistent with the location of key binding residues (Figure 7E).

## 4 Discussion

This study systematically characterized gut microbiota, fecal SCFA profiles in UC, CD, and healthy controls through multi-omic analysis. The results revealed reduced gut microbiota diversity in IBD patients compared with healthy individuals, alongside distinct microbial signatures between UC and CD. The shared elevation of 2-HB and isocaproic acid in both disease groups suggests altered branched-chain amino acid fermentation by dysbiotic microbiota (20, 21).

Cluster analysis demonstrated depletion of *Clostridiales* and Lachnospiraceae in IBD groups, both members of the phylum *Firmicutes*. These taxa are associated with gut homeostasis maintenance, potentially explaining reduced SCFA production (22, 23). Conversely, increased *Ruminococcus* abundance in UC may contribute to pro-inflammatory metabolite generation. LEfSe analysis identified several disease-specific bacteria as candidate biomarkers for discriminating IBD subtypes, including *Dialister_invisus* in CD and *Streptococcus_anginosus* in UC. These findings require validation in larger cohorts.

Regarding SCFA profiles, CD patients exhibited marked up-regulation of branched-chain SCFAs, specifically isovaleric acid and isocaproic acid. UC patients showed mixed alterations: butyric acid and valeric acid were down-regulated, while three other acids were up-regulated. The reduction of valeric acid and butyric acid in UC aligns with observed *Clostridiales* depletion. These findings suggest that disease-specific metabolic dysregulation is potentially linked to altered microbial fermentation pathways (24).

2-HB is chemically distinct from classical SCFAs, being a short-chain hydroxy acid (α-hydroxybutyrate) rather than a straight-chain carboxylic acid. While its microbial origin and colonic abundance motivate parallel investigation with SCFAs, we emphasize that its distinct ionization behavior and metabolic fate preclude direct functional equivalence. Innovatively, the concentration of 2-HB correlated positively with *Veillonella* in UC, and negatively with *Clostridium_sp._CAG:567, Firmicutes_bacterium_CAG:460*, and *Holdemanella biformis* in CD. Intestinal colonization of *Veillonella* promotes *Clostridioides difficile* infection in CD (25). The parallel association between *Veillonella* and 2-HB in UC aligns with their disease-exacerbating characteristics. This association may imply a specific role for 2-HB in IBD pathobiology. Notably, 2-HB originates from threonine and methionine catabolism, as well as glutathione synthesis under oxidative stress, and has been implicated as a marker of oxidative stress (26). Therefore, the elevation of the concentration of 2-HB in IBD might reflect a convergence of microbial dysbiosis and host redox imbalance. This elevation likely reflects increased oxidative stress in the gut during active inflammation, which drives 2-HB accumulation via glutathione turnover. Correlation analysis confirmed microbiota-SCFA interactions as critical modulators of IBD pathogenesis. Accordingly, these delicate causal directionalities all warrant further mechanistic investigation.

BKMR analysis revealed dose-response relationships between SCFAs and IBD risk. Concentrations of 2-HB and isocaproic acid were positively associated with IBD risk. Molecular docking and MD simulations identified HCN2 as a potential target for 2-HB, provided structural evidence for stable complex formation.

Ensemble-based target prediction ranked HCN2 as the top candidate, exhibiting balanced performance across druggability and CPI metrics. This hyperpolarization-activated cyclic nucleotide-gated potassium channel, predominantly expressed in the brain and heart, regulates neuronal excitability and cardiac pacemaking (27–30). Molecular dynamics simulations of the HCN2-2-HB complex validated the predicted interaction. The modeling revealed that 2-HB binds to a specific allosteric pocket of HCN2 with high stability, driven primarily by hydrophobic interactions and electrostatic anchoring via key arginine residues. This binding mode provides the structural basis for stable complex formation between 2-HB and HCN2, consistent with short-chain fatty acid-protein interactions (31, 32). These findings suggest that 2-HB may function as a modulator of HCN2 channel activity, potentially influencing cardiac pacemaking or neuronal excitability. This mechanism aligns with emerging evidence that gut microbiota-derived metabolites signal to distant organs through direct protein interactions, supporting the gut-heart and gut-brain axis concepts. However, the interaction generates a mechanistic hypothesis, but lacks functional evidence for HCN2 in intestinal inflammation. Future validation should employ site-directed mutagenesis of key residues, particularly Arg659, Arg618, and Arg617, combined with electrophysiological recordings to confirm functional impacts on HCN2 channel kinetics. Extended simulation timescales and enhanced sampling methods could reveal rare conformational events and improve binding free energy estimates (33). These analyses provide atomistic insights into the metabolite's potential modulatory effects on HCN2 function. This finding is particularly intriguing given the emerging recognition of 2-HB as a signaling molecule beyond its traditional role as an energy substrate.

The inclusion of established targets such as PARP1 and PPARA among the top tier serves as positive controls validating our predictive approach. Future directions include experimental validation through patch-clamp electrophysiology and investigation of HCN2 modulation in disease contexts characterized by altered ketone body metabolism.

Several limitations of this study should be acknowledged. The cross-sectional design, restricted to patients with active disease, prevented assessment of 2-HB levels across varying disease activity states and precluded any direct causal inference. The fecal metabolite concentrations were unable to accurately reflect mucosal levels at the site of inflammation. Moreover, the interaction between HCN2 and 2-HB lacked experimental validation. In clinical settings, the fecal SCFA profile offers an effective non-invasive monitoring approach. However, the prospective validation of diagnostic thresholds and treatment response dynamics is required. Further work is needed to provide functional evidence for HCN2 in intestinal inflammation.

In summary, multi-omic analysis identified distinct microbial-SCFA signatures in UC and CD, with 2-HB emerging as a shared risk-associated metabolite. Network pharmacology and molecular dynamics simulation suggested that 2-HB potentially acts through HCN2 modulation, providing structural evidence for complex formation. This integrative computational framework generates a mechanistic hypothesis. These findings support the development of specific fecal SCFAs as non-invasive activity markers. Future studies are needed to link specific microbial metabolites to host targets in IBD pathogenesis.

## Data Availability

The data supporting this study are not publicly available due to ethical restrictions and participant privacy protections. Deidentified data may be available from the corresponding author upon formal request and with appropriate ethical approval.
